# When Low Independence Fuels Luxury Consumption: Uniqueness as a Defense Mechanism During Collective Threats

**DOI:** 10.3390/bs15121735

**Published:** 2025-12-15

**Authors:** Jaeseok Yook, Seunghee Han

**Affiliations:** Department of Business Administration, Chung-Ang University, Seoul 06974, Republic of Korea

**Keywords:** luxury consumption, self-construal, need for uniqueness, terror management theory, collective crisis

## Abstract

Global crises, from pandemics to geopolitical instability, intensify societal anxiety. Paradoxically, these periods of collective threat often witness surges in luxury consumption. Drawing on Terror Management Theory (TMT), we propose this behavior is a psychological response to the deindividuating nature of such threats. We argue that a collective crisis increases intentions to purchase luxury goods via an intensified need for uniqueness, which functions as a self-affirming mechanism against a threatened sense of personal identity. We test this model using the COVID-19 pandemic as a salient operationalization of a collective threat. We further propose that this effect is counterintuitively moderated by independent self-construal. Findings from an experimental study (*N* = 276) show that perceived crisis risk increases luxury purchase intention, and this effect is serially mediated by the need for uniqueness. Critically, this indirect effect is strongest for individuals low in independent self-construal, who are prompted to engage in compensatory uniqueness-seeking when their primary buffer of social connection is disrupted. Our findings contribute to consumer behavior research by identifying a novel psychological pathway linking collective threats to consumption and offer insights for brands navigating consumer behavior during periods of widespread uncertainty.

## 1. Introduction

Global crises—whether pandemics, natural disasters, or geopolitical instability—heighten uncertainty and amplify societal anxiety. Yet, paradoxically, such periods frequently witness increases in luxury consumption—a behavior seemingly at odds with the collective restraint crises might be expected to inspire. The COVID-19 pandemic, with its potent combination of mortality salience and enforced social conformity, serves as a powerful recent example of such a threat, with some markets seeing record luxury sales (Kumar, 2023; Hati et al., 2024). Understanding why consumers turn to high-status goods when facing collective existential threat is both theoretically intriguing and practically important.

Drawing on Terror Management Theory (TMT; Greenberg et al., 1986), we propose that consumers under collective threat may seek to affirm their individuality through the marketplace (Arendsen et al., 2025). Specifically, we argue that perceived crisis risk increases intentions to purchase luxury goods via an intensified need for uniqueness—defined as the desire to differentiate the self from others (Snyder & Fromkin, 1977). Uniqueness serves as a self-affirming resource, helping consumers symbolically assert personal identity in the face of mortality-related anxiety. The need for uniqueness is a well-documented driver of luxury consumption (Dubois & Laurent, 1994; Bian & Forsythe, 2012). However, its function as a psychological defense mechanism against the deindividuating nature of collective existential threats has been largely overlooked, a gap this research aims to fill.

We further suggest that this effect is moderated by independent self-construal (Markus & Kitayama, 1991). Under crisis conditions, we find that the drive for uniqueness is amplified most among those low in independence—individuals who ordinarily do not define themselves in individualistic terms (Singelis, 1994). For these consumers, a collective crisis disrupts their primary source of self-worth—social connection and interdependence—prompting a compensatory shift toward self-enhancement through uniqueness. In contrast, those high in independent self-construal already possess a strong, stable sense of individuality, reducing the need to seek uniqueness through consumption under threat. This counterintuitive pattern challenges prevailing assumptions about who engages in identity-affirming consumption and when.

Our research makes three contributions: First, we extend TMT by identifying need for uniqueness as a key psychological mechanism linking collective threat to luxury consumption. Second, we demonstrate that self-construal shapes this coping process in unexpected ways, revealing that low independence can heighten identity-affirming consumption under threat. Third, we offer practical insight for marketers seeking to design culturally sensitive, crisis-aware strategies that resonate with consumers whose sense of self is unsettled.

## 2. Literature Review and Hypothesis Development

### 2.1. Collective Threats, Mortality Salience, and Terror Management Theory

Collective crises, such as pandemics or public safety emergencies, represent potent and persistent reminders of human mortality. Risk perception—an individual’s subjective assessment of potential harm (Slovic, 1987)—is central to understanding responses to such crises. In this context, perceived risk is not merely a statistical calculation but a vivid, ongoing reminder of personal vulnerability and mortality (Pyszczynski et al., 2021).

According to Terror Management Theory (TMT), this awareness of death generates existential anxiety that must be managed to preserve psychological stability (Greenberg et al., 1986). TMT posits that since literal immortality is impossible, humans strive for symbolic immortality—a sense of lasting significance achieved by living up to the standards of a valued cultural worldview. A primary vehicle for achieving this is the maintenance of self-esteem, which serves as an indicator that one is a valuable contributor to that meaningful reality (Harmon-Jones et al., 1997).

In consumerist societies, material acquisition is a culturally sanctioned means of affirming self-worth (Song et al., 2024) and pursuing symbolic immortality (Arndt et al., 2004). Luxury goods, as culturally recognized symbols of success, permanence, and transcendence, are particularly powerful tools in this process. By acquiring objects that are perceived to be of lasting value and high status, individuals can bolster their self-esteem and create a symbolic buffer against feelings of mortality. Furthermore, recent research suggests that mortality salience invokes fantastical thoughts and feelings, driving consumers toward luxury products not just for status, but as a form of hedonic escape from existential anxiety (Geiger-Oneto & Shehryar, 2025). This theoretical link is well-supported by extensive empirical work, including a recent systematic review and meta-analysis, demonstrating that mortality salience increases materialism and the consumption of status-oriented products (Arendsen et al., 2025; Mandel & Heine, 1999; Kasser & Sheldon, 2000). We therefore predict the following:

**Hypothesis** **1:** *Higher perceived risk from a collective threat will increase consumers’ intentions to purchase luxury products*.

### 2.2. The Mediating Role of the Need for Uniqueness

While TMT describes a general drive to bolster self-esteem, the specific form that this drive takes is shaped by the nature of the threat. A collective crisis like a pandemic poses a unique psychological problem: the threat of deindividuation. The constant emphasis on shared fate (“we’re all in this together”) and uniform behavioral mandates (e.g., masking, social distancing) can create a sense of being an interchangeable member of a homogenous mass, thereby threatening one’s sense of personal identity and significance.

It is important to differentiate this pathway from other potential coping mechanisms, such as seeking “emotional comfort” or “immediate gratification” (which might predict consumption of any pleasurable good). While TMT identifies a general drive to bolster self-esteem, we propose that the need for uniqueness is the specific, operative mechanism activated by a collective threat. A pandemic’s deindividuating nature threatens personal identity, making differentiation—not just general self-worth—the primary psychological goal. Thus, the need for uniqueness functions as the specific pathway through which consumers restore self-esteem and manage this existential anxiety.

In this context, a powerful strategy for restoring self-esteem is to actively counter this sense of deindividuation by affirming one’s individuality. This reflects a fundamental tension between the need for inclusion and the need for differentiation (Brewer, 1991). When a shared threat erodes a sense of personal distinction, re-establishing that distinction becomes a primary method of restoring a sense of value and control.

Luxury products are exceptionally well-suited to satisfy this specific need. Their scarcity, exclusivity, and symbolic value serve as clear and culturally legible markers of differentiation (Dubois & Laurent, 1994). By acquiring goods that few others possess, consumers can symbolically re-establish their identity as unique individuals who are distinct from the collective. This mechanism is supported by recent findings indicating that the need for uniqueness remains a critical antecedent of luxury purchase intentions, particularly when consumers seek to signal distinctiveness through brand choices (Alfoqahaa, 2024). This act of differentiation directly serves the TMT-driven goal of bolstering self-esteem, but through a pathway specifically tailored to the deindividuating nature of the collective threat. We therefore propose the following:

**Hypothesis** **2:** *The need for uniqueness will mediate the effect of perceived risk from collective threat on intentions to purchase luxury products*.

### 2.3. The Moderating Influence of Independent Self-Construal

The link between existential threat and uniqueness-seeking is not uniform; we propose it is moderated by an individual’s self-construal—the extent to which they define themselves as independent from, or interdependent with, others (Markus & Kitayama, 1991). Individuals high in independent self-construal view themselves as autonomous and unique, deriving self-esteem from expressing internal attributes. In contrast, those low in independence (i.e., high in interdependence) derive self-worth primarily from maintaining social harmony, fulfilling roles, and fostering connections (Singelis, 1994).

Here, we predict a counterintuitive moderation effect. The logic rests on the principle of compensatory consumption, where individuals consume to cope with self-discrepancies or threats to a valued aspect of their identity (Rucker & Cannon, 2019; S. Kim & Rucker, 2012). Recent empirical work supports this view, demonstrating that luxury consumption during stressful life events functions as a mechanism of psychological compensation, helping consumers recover a sense of control and well-being (S. Kim & Chang, 2023).

For individuals low in independence, a collective crisis directly threatens their primary source of self-worth. The disruption of social routines, heightened interpersonal anxiety, and the breakdown of communal stability create a significant self-discrepancy: their ideal self (connected, socially embedded) feels unattainable. This identity threat triggers a compensatory psychological shift. Lacking their usual means of self-affirmation, they turn to alternative, culturally potent strategies. Asserting personal uniqueness through the consumption of scarce, high-status goods becomes an effective, albeit unconventional, method to restore a sense of personal value and agency (Mandel et al., 2017).

Conversely, individuals high in independence already possess a strong, stable, and internalized sense of individuality. This disposition acts as a “psychological immune system” (Gilbert et al., 1998), providing a ready-made buffer against the homogenizing effects of a collective threat. Because their self-worth is less contingent on social stability, they have less need to engage in compensatory behaviors aimed at signaling uniqueness. Their identity is not under the same degree of threat, reducing the urgency to seek validation through the marketplace.

Therefore, we hypothesize that the drive to affirm uniqueness in the face of a collective threat will be most acute among those whose primary, interdependent self-concept is most threatened by the crisis:

**Hypothesis** **3:** *Independent self-construal will moderate the effect of perceived risk from a collective threat on the need for uniqueness, such that the effect is stronger for consumers with lower independent self-construal*.

### 2.4. The Conceptual Model

**Figure 1:**
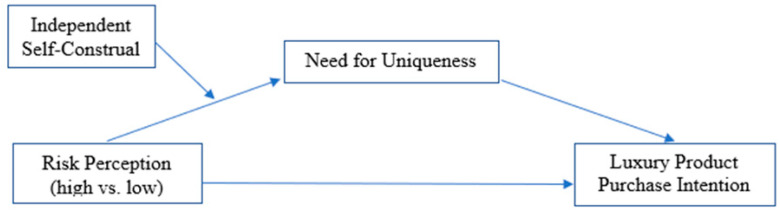
Conceptual model of hypothesized relationships.

Figure 1 presents our proposed moderated mediation model. We theorize that higher perceived risk from a collective threat will increase consumers’ intentions to purchase luxury products (H1). This effect will occur because heightened risk perception triggers a stronger need for uniqueness (H2), which functions as a psychological coping mechanism in the face of existential threat. Furthermore, we propose that independent self-construal will moderate the first stage of this pathway (H3): the effect of perceived risk on the need for uniqueness will be stronger for consumers low in independent self-construal than for those high in it. This leads to our final, integrated hypothesis:

**Hypothesis** **4:** *Independent self-construal will moderate the indirect effect of perceived risk from a collective threat on luxury purchase intention through the need for uniqueness. Specifically, the indirect effect will be significant and positive for consumers with low independent self-construal but non-significant for consumers with high independent self-construal*.

This framework integrates TMT with uniqueness-driven consumption research, extending both by identifying the following: (a) the need for uniqueness as a specific self-esteem–restoration process activated during collective crises, and (b) self-construal as a boundary condition that produces a counterintuitive pattern—where low-independence consumers exhibit the strongest uniqueness-driven luxury intentions under threat.

## 3. Materials and Methods

### 3.1. Participants

Participants were 276 residents of South Korea recruited via an online survey platform in December 2021, during a period of ongoing pandemic-related uncertainty (138 males, 50.0%; 138 females, 50.0%). The age distribution was as follows: 20–29 years (29.0%), 30–39 years (33.7%), 40–49 years (23.9%), 50–59 years (10.9%), and 60 years or older (2.5%). Monthly income varied, with the most common bracket being KRW 2–3 million (approximately USD 1500–2250; 29.7%). Eligibility criteria required participants to have purchased a luxury product within the last two years. Four screening questions assessed purchase frequency, overall satisfaction, and repurchase intentions. The most common purchase frequency over the past two years was two times (43.5%), followed by one time (23.6%). Satisfaction with the most recent luxury purchase was predominantly Satisfied (68.8%), and the same was true for average satisfaction across all past luxury purchases (65.6%). A large majority of participants (81.2%) indicated an intention to repurchase luxury goods in the future.

### 3.2. Procedure and Experimental Manipulation

This study employed a single-factor, between-subjects design (COVID-19 risk: high vs. low). After providing informed consent, participants were randomly assigned to one of two conditions in which they read a detailed scenario accompanied by images from real news sources. These materials were designed to manipulate perceived COVID-19 risk.

In the high-risk condition, participants read a vignette describing a worsening pandemic in which dangerous new viral mutations were causing exponential increases in confirmed cases and fatalities, including among healthy young adults. The scenario emphasized that developing a definitive cure would be difficult for at least a decade and predicted that mask-wearing would remain a permanent necessity.

In the low-risk condition, participants read that the pandemic was effectively over due to the successful development of proven treatments and high vaccination rates, which led to COVID-19 being reclassified as a mild illness comparable to the common cold. The scenario stated that masks were no longer needed and that large-scale public events such as festivals and sports competitions had resumed.

After reading their assigned scenario, participants were asked to imagine the emotions and feelings that they would experience in that situation. They then completed a questionnaire that included the manipulation check, measures of the dependent variable (luxury purchase intention), the mediator (need for uniqueness), the moderator (independent self-construal), and demographic questions. The demographic variables did not predict significant variance in the dependent variable (nor did it interact with any other independent variable to predict the dependent variable).

### 3.3. Measures

All multi-item measures used a 7-point Likert-type scale (1 = Strongly Disagree, 7 = Strongly Agree), unless otherwise noted. The reliability of each scale was assessed using Cronbach’s alpha. The measures used were as follows:

- Perception of COVID-19 Risk (Manipulation Check). To verify the effectiveness of the experimental manipulation, participants’ perceived risk was measured using eighteen items adapted from established risk perception scales (Slovic, 1987) and the Coronavirus Crisis Perception Scale (Stadler et al., 2020), following modifications for the COVID 19 context by Han and Lee (2021) and Yoon et al. (2021). A sample item is, “I consider COVID-19 to be a significant risk.” The scale demonstrated high reliability (Cronbach’s α = 0.979).
- Need for Uniqueness (Mediator). The need for uniqueness was measured using nine items from the Korean-Consumer’s Need for Uniqueness (K-CNFU) scale developed by W. S. Kim and Yoo (2003), which adapted Tian et al. (2001) original scale for the South Korean context. A sample item is, “I tend to seek products that highlight my style.” The scale showed excellent reliability (Cronbach’s α = 0.931).
- Independent Self-Construal (Moderator). The 6-item interdependent self-construal subscale developed by Park and Lee (2012), based on the work of Singelis (1994) and Escalas and Bettman (2005), was used. The scale measures independent self-construal (e.g., “My personal identity, independent of others, is very important to me”) and demonstrated good reliability (Independent: Cronbach’s α = 0.789).
- Luxury Purchase Intention (Dependent Variable). Participants were first presented with an image of dozens of representative luxury brand logos to establish a consistent frame of reference for the term ‘luxury brand.’ The intention to purchase luxury products was measured using eight items adapted from seminal scales by Baker and Churchill (1977), Parasuraman et al. (1993), and the cross-cultural luxury consumption scale by Bian and Forsythe (2012). A sample item is, “I am likely to purchase a luxury brand product in the near future.” The scale exhibited excellent reliability (Cronbach’s α = 0.948).

## 4. Results

### 4.1. Manipulation Checks

An independent-samples *t*-test confirmed that the experimental manipulation of COVID-19 risk perception was successful. Participants exposed to the high-risk scenario reported significantly higher perceived risk (*M* = 5.94, *SD* = 0.97) than those in the low-risk scenario (*M* = 4.13, *SD* = 1.84), *t*(274) = 10.21, *p* < 0.001. Descriptive statistics for other key variables are presented in Table 1.

### 4.2. Hypothesis Testing

#### 4.2.1. Direct Effect of Risk Perception on Luxury Purchase Intention

Hypothesis 1 predicted that higher perceived COVID-19 risk would increase intentions to purchase luxury products. An independent-samples *t*-test supported this prediction: participants in the high-risk condition reported significantly stronger purchase intentions (*M* = 5.26, *SD* = 0.99) than those in the low-risk condition (*M* = 4.29, *SD* = 1.46), *t*(274) = 6.38, *p* < 0.001.

#### 4.2.2. Mediating Role of Need for Uniqueness

Hypothesis 2 proposed that the need for uniqueness would mediate the relationship between the risk perception and luxury product purchase intention. We tested the mediation hypothesis using IBM SPSS Statistics (Version 28) with the PROCESS macro (Version 5.0; Hayes, 2018) Model 4 with 5000 bootstrap samples. The results support the proposed mediation model.

First, the path from the risk manipulation to the need for uniqueness was significant. Participants in the high-risk condition reported higher levels of need for uniqueness compared to those in the low-risk condition, *B* = 0.47, *SE* = 0.16, *t*(274) = 2.92, *p* = 0.004, 95% *CI* [0.15, 0.79]. Next, need for uniqueness significantly predicted purchase intention while controlling for the risk manipulation, *B* = 0.20, *SE* = 0.06, *t*(273) = 3.67, *p* < 0.001, 95% *CI* [0.09, 0.31].

Crucially, the analysis revealed a significant indirect effect of the risk manipulation on luxury purchase intention through the need for uniqueness, *B* = 0.10, *SE* = 0.05, 95% *CI* [0.02, 0.20]. As the confidence interval does not contain zero, this indicates a significant mediation effect. The direct effect of the manipulation on purchase intention remained significant in the model (*B* = 0.87, *p* < 0.001), indicating partial mediation. The model accounted for 17% of the variance in purchase intention, R^2^ = 0.17, *F*(2, 273) = 28.03, *p* < 0.001. Therefore, Hypothesis 2 was supported.

#### 4.2.3. Moderated Mediation by Independent Self-Construal

**Figure 2:**
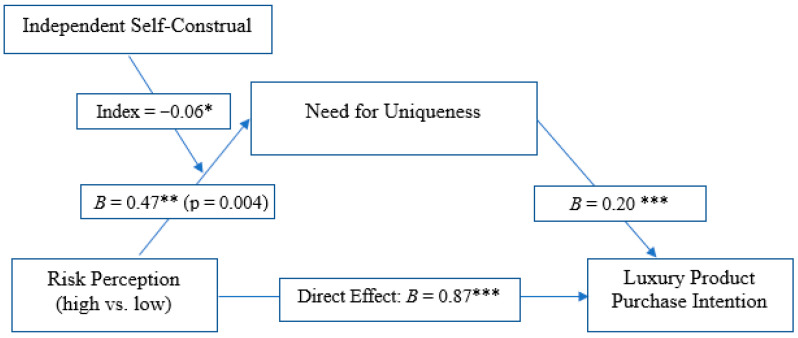
Results of moderated mediation analysis. Higher perceived risk from COVID-19 increased luxury purchase intentions both directly and indirectly through the need for uniqueness. The indirect effect of Perceived Risk on Luxury Purchase Intention through Need for Uniqueness is significant for consumers with low independent self-construal (Effect = 0.09, 95% CI [0.018, 0.1 93]) but is not significant for those with high independent self-construal. * *p* < 0.05, ** *p* < 0.01, *** *p* < 0.001.

Hypothesis 3 predicted that the mediating role of the need for uniqueness would be moderated by independent self-construal, such that the indirect effect would be stronger for individuals low in independent self-construal. This hypothesis was tested using PROCESS Model 7 with 5000 bootstrap samples. The Index of Moderated Mediation was significant (*Index* = −0.06, 95% *CI* [−0.140, −0.005]), indicating that the strength of the indirect effect varied as a function of independent self-construal (see Figure 2).

**Figure 3:**
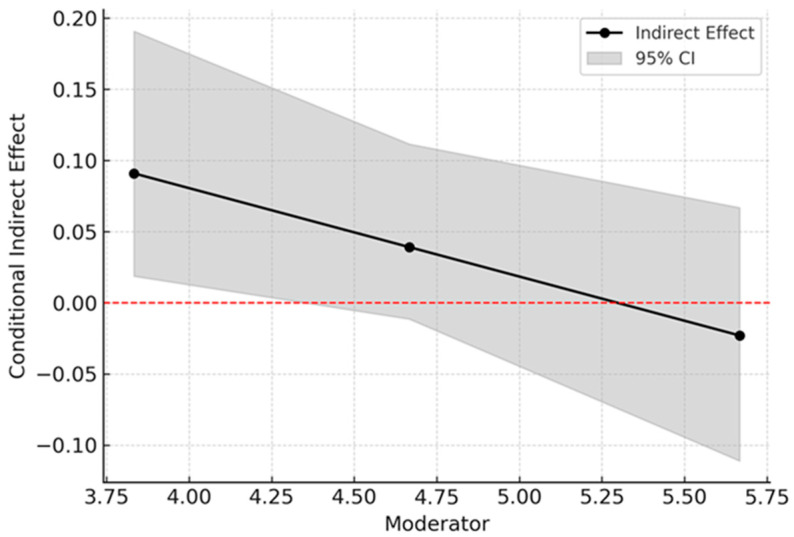
Conditional Indirect Effects at Varying Levels of Independent Self-Construal.

To probe this interaction, we examined the conditional indirect effect at three levels of independent self-construal (low, mean, and high). As predicted, for individuals with low independent self-construal (−1 *SD*), the indirect effect was positive and significant (*B* = 0.09, 95% *CI* [0.018, 0.193]). For individuals with mean independent self-construal, the indirect effect was not significant (*B* = 0.04, *SE* = 0.03, 95% *CI* [−0.014, 0.114]). For individuals with high independent self-construal (+1 *SD*), the indirect effect was also not significant (*B* = −0.02, *SE* = 0.04, 95% *CI* [−0.115, 0.065]) (See Figure 3).

That is, risk manipulation successfully increased the need for uniqueness, but only among participants who were low in independent self-construal. This suggests that using luxury purchase as a way to restore a sense of identity in the face of a collective risk is a psychological strategy primarily employed by individuals who do not define themselves through independence. Therefore, Hypothesis 3 was supported.

## 5. Discussion

### 5.1. Summary and Interpretation of Findings

This research examined how consumers respond to collective threats through the marketplace. Drawing on Terror Management Theory, we investigated whether the need for uniqueness drives luxury consumption during a crisis and how this is shaped by self-construal. Our key findings, operationalized within the context of the COVID-19 pandemic, were threefold.

First, consistent with Terror Management Theory, higher perceived risk increased luxury purchase intentions. This supports the view that consumers use high-status goods to pursue a sense of symbolic immortality, bolstering self-esteem to manage mortality-related anxiety (Arndt et al., 2004; Pyszczynski et al., 2021). Second, this effect was mediated by a heightened need for uniqueness, suggesting a specific psychological pathway. We argue that the collective and deindividuating nature of a pandemic creates a threat of deindividuation, prompting individuals to restore their sense of personal significance by differentiating themselves from the mass (Brewer, 1991). This aligns with contemporary findings indicating that the need for uniqueness remains a primary predictor of luxury purchase intention, particularly when consumers seek to assert their distinctiveness through brand choices (Alfoqahaa, 2024)

Most critically, this indirect effect was moderated by self-construal in a counterintuitive way. The drive to seek uniqueness was strongest among individuals low in independent self-construal. This finding challenges the simple assumption that individualists are always the primary seekers of uniqueness. Instead, it suggests a process of compensatory consumption (Rucker & Galinsky, 2008). For low-independence individuals, whose self-worth is rooted in social connection, a collective crisis disrupts their primary identity buffer. This triggers a compensatory shift toward uniqueness-seeking as an alternative means of restoring a threatened sense of self.

### 5.2. Theoretical Implications

Our findings contribute to the consumer behavior and social psychology literature in several key ways.

First, we extend Terror Management Theory (TMT) by identifying the need for uniqueness as a specific pathway to symbolic immortality during a collective threat. While prior TMT research links mortality salience to materialism (Mandel & Heine, 1999), our work specifies that the deindividuating nature of a shared crisis activates a powerful drive to differentiate the self as a means of restoring personal significance.

Second, our findings add critical nuance to the literature on self-construal and consumption. The prevailing view is that high-independence consumers are driven by uniqueness. Our results reveal a crucial boundary condition: under collective threat, this pattern can reverse. This demonstrates that self-construal is not a static predictor of behavior but interacts dynamically with situational threats. The loss of a primary identity buffer (social connection) can motivate low-independence individuals to adopt coping strategies that seem, on the surface, inconsistent with their dispositional tendencies.

### 5.3. Practical Implications

The findings offer actionable insights for luxury brand managers, especially in markets where interdependent self-construal is common. The consumer segment most responsive to uniqueness appeals during a crisis may not be the typical individualist, but rather those with a more relational self-concept whose social world has been disrupted. Marketers should recognize that a threat to the collective can trigger a powerful, temporary drive for individual expression.

Marketing communications should resonate with the underlying psychological tension. Instead of generic “stand out” themes, messaging can be more effective by positioning luxury as a tool for personal reaffirmation and resilience in the face of collective uncertainty. Acknowledge the shared struggle while offering a way to restore a sense of individual significance and control.

In addition, since these consumers are driven by a temporary need to counter deindividuation, brands can create powerful long-term loyalty by offering both distinction and affiliation. Exclusive membership communities or bespoke events, for example, allow consumers to signal their uniqueness while simultaneously belonging to a new, high-status in-group, satisfying both fundamental needs (Brewer, 1991).

### 5.4. Limitations and Future Research

Despite its contributions, this study has limitations that present clear avenues for future research.

Although the sample was demographically diverse, we did not analyze effects by generational cohort. Future research should examine whether the observed TMT effects differ across younger and older generations, as generational identity may influence coping strategies during collective crises. Additionally, this study’s focus on South Korean consumers limits the generalizability of findings to other cultural contexts. Future research could test whether similar patterns emerge in cultures with different independence–interdependence norms or in markets where luxury consumption carries distinct social meanings.

Second, while our study identified the ‘need for uniqueness’ as the primary psychological mediator—aligning with the deindividuating nature of the threat—it is plausible that other mechanisms operate concurrently. For instance, consumers may simply be seeking emotional comfort or immediate gratification to alleviate general anxiety, rather than specifically seeking identity differentiation. Future research could test these competing mediators to determine which psychological pathways dominate under varying threat typologies. Furthermore, whereas we argue that collective threats rupture social connections for low-independence individuals—triggering compensatory consumption—Chen (2025) found that mortality salience strengthens perceived connectedness for those with a global identity, thereby promoting prosociality. Future research should investigate how distinct layers of identity determine whether a threat disrupts or reinforces social bonds, ultimately steering coping responses toward either self-enhancement (luxury) or other-orientation (prosociality).

Third, our work operationalized the threat using the COVID-19 pandemic. This crisis was unique in that it simultaneously triggered mortality salience and enforced social isolation. The threat to low-independence individuals was thus twofold: an existential threat combined with a direct disruption of their primary coping mechanism (social connection). Future research, as suggested by Arendsen et al. (2025), should examine whether our findings replicate in other collective threats that encourage social connection, such as a national tragedy or economic recession. In such contexts, low-independence individuals might “double down” on interdependence rather than engage in compensatory uniqueness-seeking.

Fourth, this study only included consumers with prior luxury purchase experience, which ensured realism in measuring purchase intentions but limited generalizability. Future work should compare experienced and inexperienced luxury consumers to determine whether existential threats prompt market entry or trigger alternative coping behaviors.

Fifth, the findings raise questions about the coping strategies of individuals high in independent self-construal. If they are not driven to seek uniqueness under collective threat, they may engage in other TMT-related defenses that reinforce agency and competence, such as skill acquisition or financial planning. Future research could directly test for these alternative pathways, such as whether mortality salience leads high-independence consumers to invest more in skill-building or financial planning.

Finally, our study used the COVID-19 pandemic as the context for collective threat. This crisis was unique in that it simultaneously triggered mortality salience and enforced social isolation. The threat to low-independence individuals was likely twofold: an existential threat combined with a direct disruption of their primary coping mechanism (social connection). Future research should examine whether our findings replicate in other collective crises that encourage social connection, such as a national tragedy or economic recession. In such contexts, low-independence individuals might ‘double down’ on interdependence rather than engage in compensatory uniqueness-seeking.

## 6. Conclusions

This research shows that, under collective threat, the drive to affirm individuality through consumption can be strongest among those who ordinarily place less emphasis on independence. By linking TMT to uniqueness-driven luxury consumption and identifying self-construal as a counterintuitive boundary condition, we provide new insight into when and for whom luxury goods become tools for self-affirmation. In doing so, we highlight the importance of cultural–psychological nuance in understanding consumer coping strategies during times of shared uncertainty. For practitioners, the results point to clear opportunities for more nuanced, context-sensitive engagement with consumers whose identities are in flux—helping brands move beyond generic appeals toward strategies that resonate deeply with the psychological realities of crisis-driven consumption.

## Figures and Tables

**Table 1:** Descriptive statistics and mean comparisons of key variables across conditions.

	Low Risk	High Risk	*t*(274)	*p*-Value
	*M* (*SD*)	*M* (*SD*)		
Luxury Purchase Intention	4.30 (1.46)	5.26 (1.00)	6.38	<0.001
Need for Uniqueness	3.67 (1.25)	4.14 (1.41)	2.92	0.004
Independent Self-Construal	4.65 (0.81)	4.82 (1.03)	1.52	*n.s.*

*Note: M* = Mean; *SD* = Standard Deviation; *n.s.* = not significant. *n* = 138 per condition; total *N* = 276. The *t*-test for Independent Self-Construal serves as a successful check for random assignment.

## Data Availability

Data can be obtained upon request from the corresponding author.
